# Association Between Trajectories of Depressive Symptoms and Cardiovascular Disease in Elderly Chinese Adults: Findings From the China Health and Retirement Longitudinal Study (CHARLS)

**DOI:** 10.1002/brb3.70738

**Published:** 2025-08-04

**Authors:** Danhua Wang, Qiannan Chen, Apei Jiang, Xianzhen Peng

**Affiliations:** ^1^ Department of Public Health and Preventive Medicine Kangda College of Nanjing Medical University Lianyungang Jiangsu China

**Keywords:** cardiovascular disease, depressive symptom trajectories, mendelian randomization, older adults

## Abstract

**Background:**

Although the association between depressive symptoms and cardiovascular disease (CVD) has been widely investigated, most existing studies have relied on single‐timepoint assessments of depression. Examining longitudinal trajectories of depressive symptoms may provide deeper insights into the dynamic and complex relationship between depression and CVD risk.

**Methods:**

Based on data from the China Health and Retirement Longitudinal Study (CHARLS), this study analyzed the relationship between depressive symptom trajectories and CVD risk through logistic regression modeling and latent category growth modeling (LCGM) and explored causality using two‐sample Mendelian randomisation (MR).

**Results:**

The study found four depressive symptom trajectories among Chinese older adults: low‐level symptoms, symptom relief, symptom worsening, and high‐level symptoms. Compared with the low‐level symptom group, the symptom relief, symptom worsening, and high‐level symptom groups had a significantly increased risk of CVD, with ORs of 1.51, 1.60, and 2.87, respectively (all *P values* < 0.05). MR analyses also showed a significant association between depressive symptoms and CVD risk.

**Conclusions:**

The study indicates that longitudinal assessment of depressive symptom trajectories provides a more robust prediction of CVD risk compared to single‐timepoint assessments.

## Graphical abstract

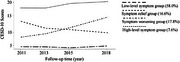

## 1 Introduction

Depression is a persistent, negative mental state characterized by sadness, frustration, a significant loss of interest and pleasure in once‐enjoyed activities, as well as a notable decline in self‐esteem and self‐worth. It is reported that depressive disorders were the 13th leading cause of overall burden and the seventh leading cause of nonfatal burden globally (GBD 2019 Diseases and Injuries Collaborators 2020; Vigo et al. 2016). WHO reported that 5.7% of the elderly population over 60 years of age have experienced depression worldwide, and in China, it is more than 10.0%. According to the China Mental Health Survey, the prevalence of major depression among the Chinese people has escalated to 3.4%, while the prevalence of depressive disorder not otherwise specified stands at 3.2% (Zhang and Ji 2019). In elderly individuals, depression predominantly affects those with chronic medical conditions and cognitive decline (Alexopoulos 2005). Depressed mood not only diminishes the quality of life among older adults, but it is also intricately linked to the aggravation of mental health concerns, a decline in cognitive abilities, and the emergence of physical health issues (Yi 2016). Several studies have shown that depressive symptoms are significantly associated with an increased risk of arthritis (He et al. 2023) and are negatively associated with quality of life in diabetic patients (Zheng et al. 2023).

Previous studies have found that depression remains a risk factor for limited physical activity, self‐rated health, and poorer overall quality of life in patients with CVD after excluding the effect of differences in cardiac functioning (Ruo et al. 2003). Some studies suggest that increased depressive symptoms are associated with an increased risk of cardiovascular disease (CVD) development (Han et al. 2021; Qiu et al. 2024); it can be argued that major depression is highly comorbid with cardiovascular disease (Han et al. 2021). However, the developmental trajectory of depressive mood exhibits significant group heterogeneity, with individuals possessing diverse characteristics potentially manifesting unique patterns of emotional decline (Kaup et al. 2016). Furthermore, it is conceivable that distinct trajectories of depressive mood could signify varying underlying causes of symptoms and potentially exert diverse impacts on patient outcomes (Mirza et al. 2016).

In comparison to the extensive research exploring the association between depression and CVD, there exists a relatively scant number of studies delving into the relationship between the trajectories of depressive change and CVD. Therefore, the study employed the latent class growth model (LCGM) to investigate the influence of trajectories of depressive mood changes on the risk of developing cardiovascular disease among older adults.

## 2 Materials and Methods

### 2.1 Study Population

Our study is a longitudinal study based on data from the China Health and Retirement Longitudinal Study (CHARLS), which aims to investigate the social, economic, and health status of middle‐aged and elderly people over 45 in China (Zhao et al. 2012). The national baseline survey (Wave 1), including 17,708 participants, was conducted in 2011. Wave 2, Wave 3, and Wave 4 followed in 2013, 2015, and 2018. Exclusion criteria were (i) lost to follow up and missing data on cognitive function (n = 10229) and (ii) individuals with CVD at baseline (1003). Ultimately, a total of 6,476 participants were included in the study. The ethical committee of Peking University approved the CHARLS (IRB00001052‐11015). All participants have submitted a written informed consent.

### 2.2 Depressive Symptoms Ascertainment

The depressive symptoms were assessed using the 10‐item Center for Epidemiological Studies Depression Scale (CES‐D‐10) (Björgvinsson et al. 2013). The scale comprises a total of 10 items, designed to assess the feelings and behaviors of the study subjects over the past week. Each item reflects a varying degree of depression, ranging from low to high, and is assigned a corresponding value of 0–3 points accordingly. The maximal total score is 30, with higher scores reflecting greater symptoms and a score of 10 or higher indicating clinically significant depression.

### 2.3 CVD Ascertainment

According to the questions in the CHARLS follow‐up questionnaire, such as “Has a doctor told you that you have had a heart attack (myocardial infarction, coronary heart disease, angina, congestive heart failure, or other heart problems) since your last visit?” and “Has a doctor told you that you have had a stroke since your last visit?”, any one of the questions should be answered “yes.” A “yes” answer to either question was considered a CVD outcome.

### 2.4 Covariates Assessed in 2011–2018

Demographic and sociological information including age, gender, height, weight, body mass index (BMI), education level, marital status, smoking status (yes or no)—“smoking” means that the respondent reported smoking at some point, and “no smoking” means that the respondent reported never having smoked—drinking status (never, <1 time /month, ≥1 time/month), educational level (primary and below, primary and above), and whether they have other chronic diseases (diabetes, hypertension, physical disability, dyslipidemia, and so on).

## 3 Statistical Analysis

### 3.1 Trajectories of Depression Symptoms

We utilized the statistical procedure SAS Proc Traj from SAS Institute Inc. to conduct a latent class growth curve analysis, aiming to accurately estimate the mean trajectories of CES‐D‐10 scores across various visits, with scores being measured in the years 2011, 2013, 2015, and 2018. LCGM was applied using linear and quadratic polynomials with three to five trajectory categories (individuals per trajectory ≥1%), and the model with the highest number of fitting categories was selected using the Bayesian Information Criterion (BIC) method and the average posterior probability (AvePP) of each trajectory (Tasdelen et al. 2011). The optimal model was selected based on the following criteria: (i) for each trajectory group, obtaining a close correspondence between the estimated probability of group membership and the proportion assigned to that group based on the posterior probability of group membership; (ii) ensuring that the average of the posterior probabilities of group membership for individuals assigned to each group exceeded a minimum threshold of 0.7; (iii) establishing that the odds of correct classification based on the posterior probabilities of group membership exceeded a minimum threshold of 5; and (iv) observing reasonably tight confidence intervals around the estimated probabilities of group membership.

We compared covariates across the observed trajectories using analysis of variance (ANOVA) for continuous and chi‐square tests for categorical variables. A binary logistic regression model was used to analyze the association between trajectories of depressive symptoms and CVD. We also did several sensitivity analyses to examine the reliability of the results. First, previous studies have shown that there is a causal relationship between smoking, drinking, and depressive symptoms, and there is also a significant association with CVD (Boden and Fergusson 2011; Lee et al. 2023). Therefore, in order to further control the impact of smoking and drinking, we performed subgroup analysis to evaluate the heterogeneity of the association between depression symptom trajectories and CVD risk among the non‐smoking and non‐drinking individuals. Second, two‐sample Mendelian randomization (MR) analysis was also performed to assess the causality between depression and CVD risk, including inverse‐variance weighted (IVW), Mendelian randomization Egger (MR Egger), and so on. We selected genetic instruments (SNPs) for depression from the GWAS by Howard et al. (2019), which included 246,363 cases and 561,190 controls. SNPs were extracted at a genome‐wide significance threshold (*P* < 5×10^−8^) and clumped (r^2^ < 0.001, kb = 10,000) to ensure independence.

All tests were 2‐tailed, and the level of significance was set at 0.05. Analyses were performed using R software (version 4.2.1).

## 4 Results

### 4.1 Trajectories of Depressive Symptoms Among Older Adults, 2011–2018

**FIGURE 1:**
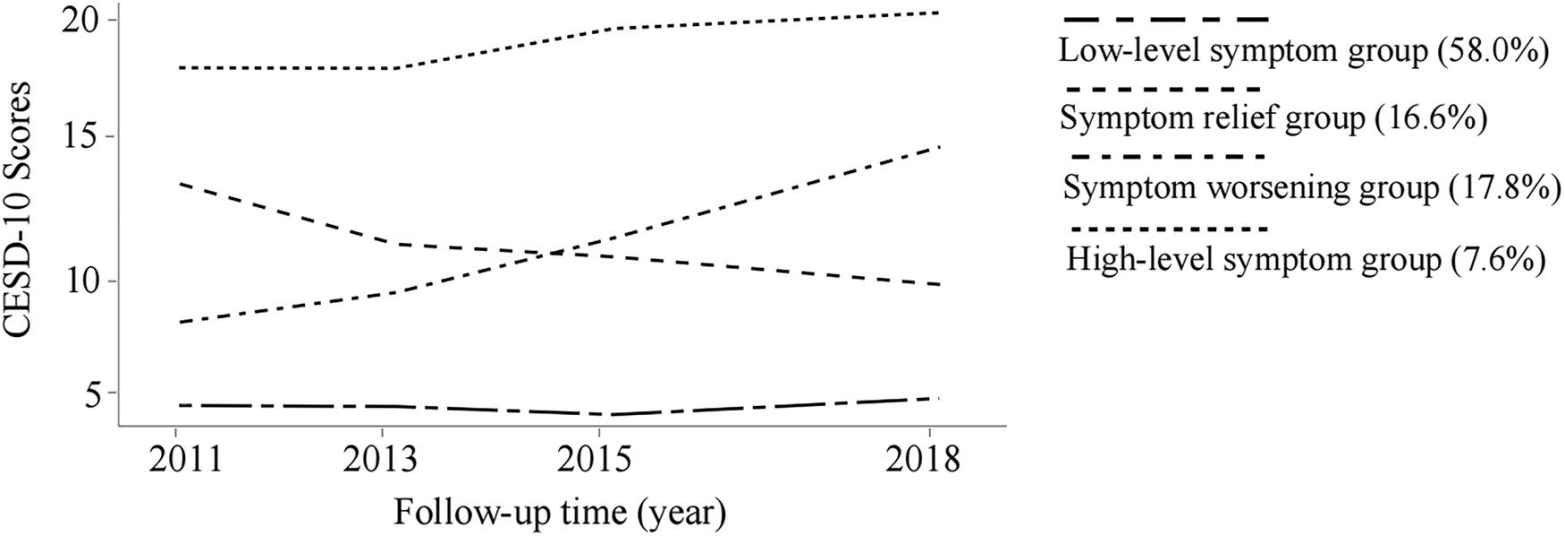
Trajectories of depressive symptoms among the CHARLS study participants.

We identified four distinct depressive symptom trajectories by the LCGM (Figure 1). Depressive symptom trajectories of 6,476 researchers were optimal when estimated into 4 categories by LCGM (BIC = ‐76909.50): the low‐level symptom group (N = 3,906, 58.0%), the symptom relief group (N = 986, 16.6%), the symptom worsening group (N = 1,090, 17.8%), and the high‐level symptom group (N = 494, 7.6%).

Four CESD‐10 measurements were obtained at Wave 1 (2011–2012), Wave 2 (2013–2014), Wave 3 (2014–2015), Wave 4 (2017–2018) and were used to fit trajectories.

### 4.2 Baseline Characteristics of the Subjects

The basic characteristics of the subjects with the four trajectory groups are presented in Table 1. As summarized in Table 1, age, BMI, gender, education, marital status, smoking, drinking rate, physical disability and CVD differed across trajectory groups (all *p* values < 0.05).

**TABLE 1:** Participant characteristics by depressive symptom trajectory group in the CHARLS database.

Variables	Depression trajectories subgroup				*p* value
	Low‐level symptoms(N = 3,906)	Symptom relief(N = 986)	Symptom worsening(N = 1,090)	High‐level symptoms(N = 494)	
Age (y)	56.2(8.0)	56.9(8.0)	56.2(8.0)	56.9(8.1)	0.042
BMI (kg/m^2^)	23.83(3.47)	23.13(3.63)	23.57(3.61)	23.18(3.68)	<0.001
Gender(N,%)					<0.001
male	1,899 (56.0)	384 (43.0)	384 (39.0)	120 (27.0)	
female	1,463 (44.0)	500 (57.0)	595 (61.0)	320 (73.0)	
Education(N,%)					<0.001
Primary and below	1,887 (56.0)	646 (73.0)	696 (71.0)	369 (84.0)	
Primary and above	1,478 (44.0)	239 (27.0)	284 (29.0)	71 (16.0)	
Marital status(N,%)					<0.001
Spouse‐less	217 (6.4)	88 (9.9)	68 (6.9)	72 (16.0)	
Spousal	3,148 (93.6)	797 (90.1)	912 (93.1)	368 (84.0)	
Smoking rate(N,%)					<0.001
no	1,910 (57.0)	562 (64.0)	633 (65.0)	322 (73.0)	
yes	1,455 (43.0)	323 (36.0)	347 (35.0)	118 (27.0)	
Diabetes(N,%)					0.300
no	3,213 (96.1)	835 (95.1)	926 (95.2)	421 (96.3)	
yes	129 (3.9)	43 (4.9)	47 (4.8)	16 (3.7)	
Dyslipidemia(N,%)					0.800
no	3,076 (92.7)	808 (92.8)	897 (93.5)	404 (93.1)	
yes	244 (7.3)	63 (7.2)	62 (6.5)	30 (6.9)	
Hypertension(N,%)					0.200
no	2,758 (82.0)	702 (80.0)	781 (80.0)	352 (80.0)	
yes	598 (18.0)	177 (20.0)	195 (20.0)	86 (20.0)	
Drinking rate(N,%)					<0.001
never	2,030 (60.1)	608 (68.7)	696 (71.1)	326 (74.1)	
<1 time /month	1,037 (31.0)	212 (24.0)	213 (21.7)	76 (17.3)	
≥1 time /month	298 (8.9)	65 (7.3)	71 (7.2)	38 (8.6)	
Physical disability(N,%)					<0.001
no	3,282 (97.5)	843 (95.3)	944 (96.3)	417 (94.8)	
yes	83 (2.5)	42 (4.7)	36 (3.7)	23 (5.2)	
CVD(N,%)					<0.001
no	2,861 (90.8)	700 (87.0)	766 (86.0)	299 (78.0)	
yes	290 (9.2)	106 (13.0)	124 (14.0)	83 (22.0)	

*Note*: Data are presented as mean (standard deviation) for quantitative data and n (%) for qualitative data.

### 4.3 Association Between Depressive Symptom Trajectory Group and Risk of CVD

Results of unadjusted and adjusted binary logistic regression models are shown in Table 2. In models 1 and 2, compared to the low‐level symptom group, participants in the second category of symptom relief group had a significantly increased risk of CVD (adjusted OR = 1.51, 95% CI = 1.18‐1.92); the third category of symptom worsening group had a 60% increased risk of CVD compared with that of the low‐level symptom group (adjusted OR = 1.60, 95% CI = 1.26‐2.02); and the fourth category of high‐level symptoms group had 2.87 times higher risk of CVD than the low‐level symptoms group (adjusted OR = 2.87, 95% CI = 2.15‐3.80).

**TABLE 2:** Association between depressive symptom trajectory group and risk of CVD among 6,476 older adults.

Depression symptom trajectory group	Odds ratio (95%CI)^a^	
	Model 1	Model2
Low‐level symptoms	1[Reference]	1[Reference]
Symptoms relief	1.49(1.17,1.89)	1.51(1.18,1.92)
Symptoms worsening	1.60(1.27,1.99)	1.60(1.26,2.02)
High‐level symptoms	2.74(2.08,3.58)	2.87(2.15,3.80)

^a^ Model 1 is unadjusted. Model 2 is adjusted for age, gender, BMI, smoking, drinking, dyslipidemia, hypertension, diabetes, and physical disability.

### 4.4 Sensitivity Analysis

Sensitivity analysis was conducted to exclude the influence of smoking and alcohol consumption on our study. The results shown in Supplementary Tables 1 and 2 are similar to our primary findings. Furthermore, when considering the correlation between depressive symptom trajectory groups and CVD among subpopulations stratified by age, gender, education, marital status, smoking habits, drinking patterns, diabetes mellitus, dyslipidemia, hypertension, and physical disability, the results consistently demonstrated a notable elevation in the risk of CVD among individuals experiencing a higher degree of depressive symptom progression and severity, as evident in Supplementary Table 3. To assess the potential effect of unmeasured confounders, we further supplemented the E‐value analysis (VanderWeele and Ding 2017). The minimum effect size (OR = 1.51) observed between the four depression trajectories and CVD in this study corresponds to an E‐value of 1.76. Per VanderWeele's method, this E‐value (1.76) indicates that an unmeasured confounder would need to be associated with both the exposure and outcome at risk ratios of at least 1.76 each to fully explain away the observed association.

Two‐sample Mendelian randomization (MR) analysis showed that the causal association between depression and CVD risk was also consistent with our primary results, including weighted median (*β* = 0.568, *p* value < 0.05) and inverse variance weighted method (*β* = 0.425, *p* value < 0.05). The robustness of the results was confirmed by the leave‐one‐out sensitivity test (Supplementary Figure 1). The forest plot and scatter plot of causal relationships between depression and the risk of CVD are shown in Supplementary Figures 2 and 3. No directional pleiotropy was found in the MR‐Egger regression (*β* = ‐0.855, se = 1.362, *p* = 0.534). The details of two‐sample MR are shown in Supplementary Table 4. The MR‐related results were accomplished using the MRbase website (https://www.mrbase.org/).

## 5 Discussion

In our study, a total of four different trajectories of depressive mood change were identified through LCGM: low‐ level ‐ symptoms, symptom relief, symptom worsening, and high ‐ level symptoms. It was found that people in the symptom relief, symptom worsening, and high‐level symptom groups had a higher risk of CVD compared to the depressive symptom change trajectories of the low‐level symptom group. Although a large number of previous studies have suggested a clear correlation between increased depressive symptoms and increased incidence of cardiovascular events and increased risk of CVD death in Chinese adults (Han et al. 2021; Krittanawong et al. 2023; Li et al. 2019; Meng et al. 2020), fewer studies have quantified the associations between longitudinal changes in depressive symptoms and CVD events (Han et al. 2021; Han et al. 2021). This study not only utilized long‐term changes in depression to explore the effects on CVD but also utilized two‐sample MR analyses to reveal significant associations between depressive symptoms and the development of CVD. It has also been shown that higher levels of cardiovascular fitness over time are associated with a lower risk of depressive symptoms (van Sloten et al. 2023). To rigorously examine the directionality of causation, we conducted bidirectional Mendelian randomization analyses. In the reverse MR analysis specifying CVD as the exposure and depression as the outcome, we observed no statistically significant association (*p‐*value > 0.05). Conversely, our primary MR analysis demonstrated that genetic liability to depression significantly increased CVD risk. These findings align with a recent meta‐analysis of MR studies (Zeng et al. 2025) and are further supported by clinical evidence suggesting that psychological interventions for depression may reduce CVD risk (El Baou et al. 2023). Collectively, these results strengthen the evidence for depression as a causal risk factor for CVD, rather than reflecting reverse causation.

Research on the association between depressive symptoms and CVD has led to an increase in research on the mechanisms of the association, multiple potential biological and behavioral mediators have been identified, including physical inactivity (Win et al. 2011), lower heart rate variability (HRV)(Carney and Freedland 2009; Pizzi et al. 2008), inflammatory biomarkers (Carney and Freedland 2016; Harshfield et al. 2020; Pizzi et al. 2008), endothelial function (Pizzi et al. 2008), hypothalamus‐pituitary‐adrenal gland(HPA) axis hyperactivity (Wu et al. 2021), low omega‐3 fatty acid levels (Frasure‐Smith et al. 2004), antidepressant treatment (Paz‐Filho et al. 2010; Pérez‐Piñar et al. 2016), and so on. Of these, physical activity, inflammatory biomarkers, and HRV are the only candidate mechanisms shown to modulate this association (Elderon and Whooley 2013). Previous studies have also found that depression significantly affects lipid metabolism (Jung et al. 2021), and abnormalities in lipid metabolism affect the levels and distribution of multiple lipids in the body, thereby influencing the onset and progression of CVD (Soppert et al. 2020). Physical activity often benefits both depression and CVD (Reed et al. 2022; Virani et al. 2023). Physical activity and depression showed a negative correlation (Johansson et al. 2021). Physical activity was effective in reducing low‐density lipoprotein cholesterol (LDL‐c) and total cholesterol (TC), improving high‐density lipoprotein cholesterol (HDL‐c) and triglycerides, and exercise increased fitness and fat loss (Martínez‐Vizcaíno et al. 2022). Moreover, depression is recognized as a pro‐inflammatory state; some of the inflammatory markers that are elevated in depressed patients include C‐reactive protein (CRP) and interleukin 12 (IL‐12) (Osimo et al. 2020), and elevations of CRP and IL‐6 are even higher in coronary heart disease comorbid with depression (Nikkheslat et al. 2015). In addition to physical activity and inflammatory factors, HRV also affects depression and CVD. Studies have shown that all HRV measures are lower in depression than in healthy controls, and given the predictive value of HRV for cardiovascular health, reduced HRV may be one of the physiologic factors mediating the association between depression and CVD (Carney and Freedland 2009; Koch et al. 2019; Wu et al. 2023). Based on the continued interest in depressive symptoms, further exploration of the mechanisms of depression and CVD association studies is our next research focus.

This study identified the trajectories of depressive symptoms in the population based on data from a large population cohort with long‐term follow‐up, which helps to reveal the underlying biological, psychological, and behavioral mechanisms linking depressive symptoms and CVD. It helps to increase healthcare professionals' attention to patients' mental health and guides the development of preventive measures. There are some limitations to this study. First, although the results of this study were adjusted for some known confounders, some unmeasured confounders, such as genetic factors, lifestyle, and social support, may have influenced the results of this study. Second, some of the data used in this study relied on subjects' self‐reports rather than clinical diagnoses, which may create subjective bias and recall bias. Thirdly, the subjects in this study were Chinese and not representative of all ethnic populations. The analysis conducted in this study revealed a statistically significant correlation between the trajectories of depressive mood and cardiovascular disease. However, based on the available data, it was not feasible to establish a definitive causal relationship between the two phenomena. Therefore, more studies are needed to verify this. Future studies should incorporate multimodal assessments, including clinical interviews of depression and biomarkers, to further elucidate the causal relationship between depressive symptom trajectories and cardiovascular disease risk. Importantly, the depression‐CVD association may vary across individuals due to heterogeneous biological and psychological factors. Further research is needed to investigate the diverse mechanistic pathways underlying this relationship, which may involve inflammatory processes, neuroendocrine dysregulation, and behavioral mediators. Such investigations will provide a more comprehensive understanding of the complex interplay between depressive symptoms and cardiovascular health.

## 6 Conclusions

This study indicated that compared with the low‐level depressive symptoms group, the trajectories of symptom relief, symptom worsening, and high‐level depressive symptoms all significantly increased the risk of CVD. In addition, two‐sample MR using depression‐associated genetic variants revealed a similar association between depression and CVD risk. Our findings indicate that longitudinal tracking of depressive symptom trajectories is a more accurate predictor of CVD risk than single‐timepoint assessments.

## Author Contributions

**Danhua Wang**: conceptualization, funding acquisition, formal analysis, data curation, writing ‐ review and editing. **Qiannan Chen**: writing ‐ original draft, writing ‐ review and editing, validation, formal analysis. **Apei Jiang**: supervision, writing ‐ review and editing, formal analysis. **Xianzhen Peng**: conceptualization, methodology, software, data curation, writing ‐ review and editing.

## Ethics Statement

The Ethics Committee of Peking University approved the CHARLS (IRB00001052‐11015). All participants provided informed consent at the baseline assessment.

## Conflicts of Interest

The authors declare no conflicts of interests.

## Peer Review

The peer review history for this article is available at https://publons.com/publon/10.1002/brb3.70738.

## Supporting information




**Supplementary Material**: brb370738‐sup‐0001‐SuppMat.docx

## Data Availability

Data are available in a public, open‐access repository. CHARLS data of the study will be available to investigators at the CHARLS website (http://charls.pku.edu.cn/en).All GWAS summary data used in this study can be found at the “MRbase” website https://www.mrbase.org/.
