# Supplementary material for: Association Between Trajectories of Depressive Symptoms and Cardiovascular Disease in Elderly Chinese Adults: Findings From the China Health and Retirement Longitudinal Study (CHARLS)
Source: Brain Behav. 2025 Aug 4;15(8):e70738. doi: 10.1002/brb3.70738 (PMC12321971; doi:10.1002/brb3.70738)
Supplement: Supplementary file 1 — Supplementary Material: brb370738‐sup‐0001‐SuppMat.docx [file BRB3-15-e70738-s001.docx]

| Supplementary Table 1. Analysis of the relationship between trajectories of changes in depressive mood and cardiovascular disease in the non-smoking population | | | | | |
| --- | --- | --- | --- | --- | --- |
| Depression Trajectory Subgroup | *β* | *SE* | *Z value* | *OR(95%CI)* | *P value* |
| Low-level symptoms | Ref |  |  |  |  |
| Symptom relief | 0.505 | 0.158 | 3.205 | 1.66(1.21,2.25) | 0.001 |
| Symptom worsen | 0.562 | 0.149 | 3.769 | 1.75(1.31,2.35) | <0.001 |
| High-level symptoms | 1.093 | 0.177 | 6.188 | 2.98(2.10,4.20) | <0.001 |
| The ORs was adjusted for age, gender, BMI,drinking,dyslipidemia, hypertension, diabetes, physical disability | | | | | |

| Supplementary Table 2. Analysis of the relationship between trajectories of changes in depressive mood and cardiovascular disease in the non-drinking population | | | | | |
| --- | --- | --- | --- | --- | --- |
| Depression Trajectory Subgroup | *β* | *SE* | *Z value* | *OR(95%CI)* | *P value* |
| Low-level symptoms | Ref |  |  |  |  |
| Symptom relief | 0.341 | 0.151 | 2.260 | 1.40(1.04,1.89) | 0.024 |
| Symptom worsen | 0.346 | 0.145 | 2.385 | 1.41(1.06,1.87) | 0.017 |
| High-level symptoms | 1.040 | 0.170 | 6.126 | 2.83(2.02,3.93) | <0.001 |
| The ORs was adjusted for age, gender, BMI,smoking,dyslipidemia, hypertension, diabetes, physical disability | | | | | |

| Supplementary Table 3. Analysis of the association between trajectories of changes in depressed mood and cardiovascular disease under different indicator subgroups | | | | |
| --- | --- | --- | --- | --- |
| Variables |  | Depression Trajectory Subgroup | OR(95%CI) | P value |
| Age (y) | N |  |  |  |
| <60 | 2239 | Low-level symptoms | Ref |  |
|  | 560 | Symptom relief | 1.34(0.97,1.83) | 0.072 |
|  | 657 | Symptoms worsen | 1.75(1.32,2.30) | <0.001 |
|  | 267 | High-level symptoms | 2.75(1.90,3.90) | <0.001 |
| ≥60 | 1123 | Low-level symptoms | Ref |  |
|  | 325 | Symptom relief | 1.68(1.16,2.40) | 0.005 |
|  | 320 | Symptoms worsen | 1.37(0.92,2.00) | 0.108 |
|  | 172 | High-level symptoms | 2.53(1.64,3.83) | <0.001 |
| Sex |  |  |  |  |
| male | 1899 | Low-level symptoms | Ref |  |
|  | 384 | Symptom relief | 1.35(0.92,1.92) | 0.11 |
|  | 384 | Symptoms worsen | 1.76(1.24,2.46) | 0.001 |
|  | 120 | High-level symptoms | 2.82(1.69,4.52) | <0.001 |
| female | 1463 | Low-level symptoms | 1 |  |
|  | 500 | Symptom relief | 1.57(1.14,2.15) | 0.006 |
|  | 595 | Symptoms worsen | 1.45(1.07,1.96) | 0.016 |
|  | 320 | High-level symptoms | 2.58(1.84,3.61) | <0.001 |
| Education |  |  |  |  |
| Primary and below | 1887 | Low-level symptoms | Ref |  |
|  | 646 | Symptom relief | 1.67(1.25,2.22) | <0.001 |
|  | 696 | Symptoms worsen | 1.63(1.23,2.15) | <0.001 |
|  | 369 | High-level symptoms | 2.70(1.96,3.69) | <0.001 |
| Primary and above | 1478 | Low-level symptoms | Ref |  |
|  | 239 | Symptom relief | 1.16(0.72,1.81) | 0.524 |
|  | 284 | Symptoms worsen | 1.61(1.07,2.36) | 0.018 |
|  | 71 | High-level symptoms | 3.43(1.87,6.05) | <0.001 |
| Marital status |  |  |  |  |
| Spouse-less | 217 | Low-level symptoms | Ref |  |
|  | 88 | Symptom relief | 1.22(0.53,2.66) | 0.63 |
|  | 68 | Symptoms worsen | 1.37(0.57,3.08) | 0.461 |
|  | 72 | High-level symptoms | 2.17(1.00,4.59) | 0.045 |
| Spousal | 3184 | Low-level symptoms | Ref |  |
|  | 797 | Symptom relief | 1.52(1.18,1.94) | <0.001 |
|  | 912 | Symptoms worsen | 1.61(1.27,2.03) | <0.001 |
|  | 368 | High-level symptoms | 2.81(2.09,3.76) | <0.001 |
| Smoking |  |  |  |  |
| no | 1910 | Low-level symptoms | Ref |  |
|  | 562 | Symptom relief | 1.62(1.20,2.18) | 0.001 |
|  | 633 | Symptoms worsen | 1.71(1.29,2.27) | <0.001 |
|  | 322 | High-level symptoms | 2.78(1.99,3.85) | <0.001 |
| yes | 1455 | Low-level symptoms | Ref |  |
|  | 323 | Symptom relief | 1.29(0.86,1.90) | 0.206 |
|  | 347 | Symptoms worsen | 1.41(0.95,2.04) | 0.077 |
|  | 118 | High-level symptoms | 2.68(1.60,4.34) | <0.001 |
| Diabetes |  |  |  |  |
| no | 3213 | Low-level symptoms | 1 |  |
|  | 835 | Symptom relief | 1.57(1.23,2.00) | <0.001 |
|  | 926 | Symptoms worsen | 1.54(1.22,1.95) | <0.001 |
|  | 421 | High-level symptoms | 2.71(2.04,3.57) | <0.001 |
| yes | 129 | Low-level symptoms | 1 |  |
|  | 43 | Symptom relief | 0.63(0.17,1.83) | 0.427 |
|  | 47 | Symptoms worsen | 2.49(1.07,5.72) | 0.032 |
|  | 16 | High-level symptoms | 4.43(1.29,14.92) | 0.015 |
| Dyslipidemia |  |  |  |  |
| no | 3076 | Low-level symptoms | 1 |  |
|  | 808 | Symptom relief | 1.55(1.20,1.99) | <0.001 |
|  | 897 | Symptoms worsen | 1.69(1.32,2.14) | <0.001 |
|  | 404 | High-level symptoms | 3.12(2.34,4.12) | <0.001 |
| yes | 244 | Low-level symptoms | 1 |  |
|  | 63 | Symptom relief | 1.38(0.67,2.73) | 0.368 |
|  | 62 | Symptoms worsen | 1.15(0.55,2.29) | 0.695 |
|  | 30 | High-level symptoms | 0.98(0.31,2.62) | 0.976 |
| Hypertension |  |  |  |  |
| no | 2758 | Low-level symptoms | 1 |  |
|  | 702 | Symptom relief | 1.75(1.32,2.29) | <0.001 |
|  | 781 | Symptoms worsen | 1.68(1.28,2.18) | <0.001 |
|  | 352 | High-level symptoms | 2.42(1.72,3.34) | <0.001 |
| yes | 598 | Low-level symptoms | 1 |  |
|  | 177 | Symptom relief | 0.91(0.55,1.46) | 0.713 |
|  | 195 | Symptoms worsen | 1.37(0.88,2.10) | 0.159 |
|  | 86 | High-level symptoms | 4.15(2.43,7.07) | <0.001 |
| Drinking |  |  |  |  |
| never | 2030 | Low-level symptoms | 1 |  |
|  | 608 | Symptom relief | 1.38(1.03,1.84) | 0.026 |
|  | 696 | Symptoms worsen | 1.36(1.03,1.78) | 0.03 |
|  | 326 | High-level symptoms | 2.67(1.93,3.65) | <0.001 |
| ＜1 time /month | 1037 | Low-level symptoms | 1 |  |
|  | 212 | Symptom relief | 1.72(1.04,2.74) | 0.028 |
|  | 213 | Symptoms worsen | 2.51(1.60,3.86) | <0.001 |
|  | 76 | High-level symptoms | 3.18(1.67,5.76) | <0.001 |
| ≥1 time /month | 298 | Low-level symptoms | 1 |  |
|  | 65 | Symptom relief | 1.55(0.59,3.67) | 0.337 |
|  | 71 | Symptoms worsen | 1.33(0.51,3.11) | 0.533 |
|  | 38 | High-level symptoms | 1.57(0.44,4.44) | 0.434 |
| Physical disability |  |  |  |  |
| no | 3282 | Low-level symptoms | 1 |  |
|  | 843 | Symptom relief | 1.48(1.15,1.88) | 0.002 |
|  | 944 | Symptoms worsen | 1.64(1.30,2.06) | <0.001 |
|  | 417 | High-level symptoms | 2.70(2.03,3.55) | <0.001 |
| yes | 83 | Low-level symptoms | 1 |  |
|  | 42 | Symptom relief | 1.49(0.50,4.26) | 0.459 |
|  | 36 | Symptoms worsen | 0.66(0.14,2.34) | 0.549 |
|  | 23 | High-level symptoms | 2.83(0.85,8.99) | 0.08 |

| Supplementary Table 4. Sensitivity analysis of the relationship between depression trajectory and CVD using two-sample Mendelian randomization | | | | | | | |
| --- | --- | --- | --- | --- | --- | --- | --- |
| id.exposure | id.outcome | outcome | exposure | method | *β* | SE | *P* value |
| ieu-b-102 | ebi-a-GCST90086056 | Cardiovascular disease \|\| id:ebi-a-GCST90086056 | Major depression \|\| id:ieu-b-102 | MR Egger | -0.855 | 1.362 | 0.534 |
| ieu-b-102 | ebi-a-GCST90086056 | Cardiovascular disease \|\| id:ebi-a-GCST90086056 | Major depression \|\| id:ieu-b-102 | Weighted median | 0.568 | 0.251 | 0.024 |
| ieu-b-102 | ebi-a-GCST90086056 | Cardiovascular disease \|\| id:ebi-a-GCST90086056 | Major depression \|\| id:ieu-b-102 | Inverse variance weighted | 0.425 | 0.179 | 0.017 |
| ieu-b-102 | ebi-a-GCST90086056 | Cardiovascular disease \|\| id:ebi-a-GCST90086056 | Major depression \|\| id:ieu-b-102 | Weighted mode | 0.566 | 0.359 | 0.123 |
| CVD:cardiovascular disease | | | | | | | |


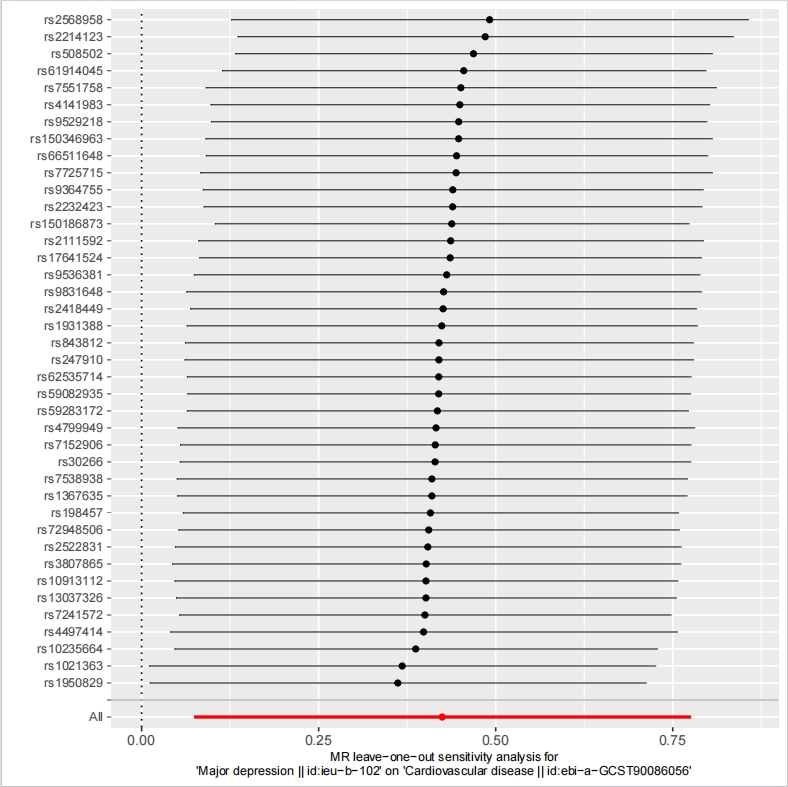


Supplementary Figure 1. Leave one out of sensitivity tests. Calculate the MR results of the remaining IVs after removing the IVs one by one.


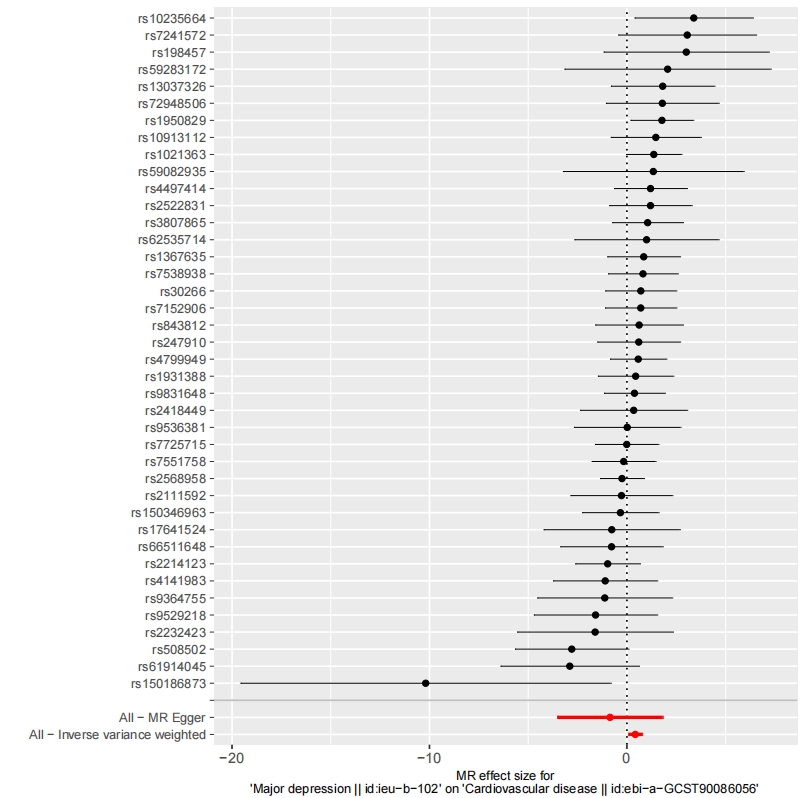


Supplementary Figure 2. Detailed Forest plots with the estimated MR effect of each IV in IVW models.


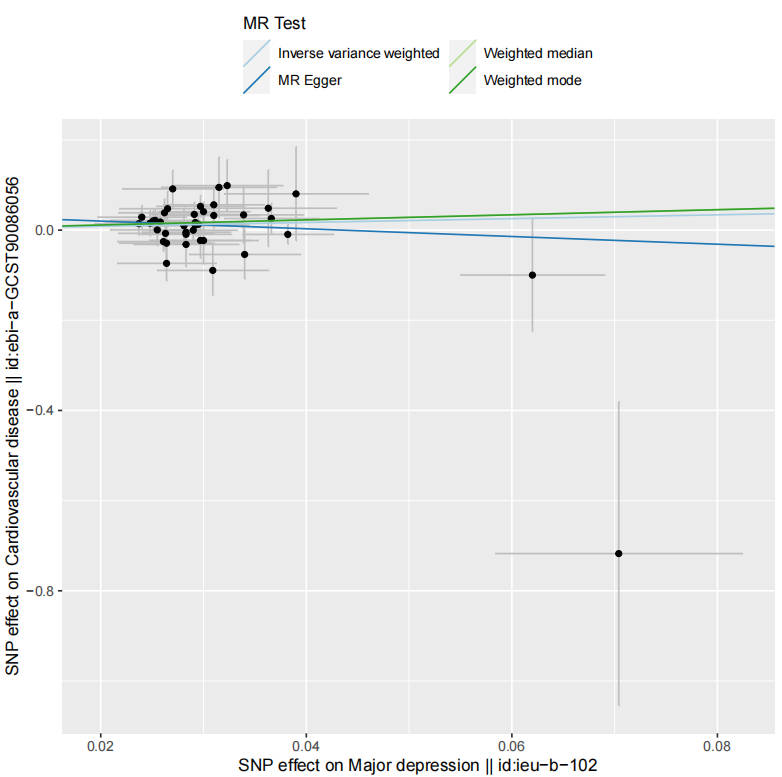


Supplementary Figure 3. Scatter plots of causality. The slope of each line corresponding to the estimated MR effect in different models.
